# Lessons from the Past: Examining the Historical Context of Trachoma Management in Iran (1925–1941)

**DOI:** 10.34172/aim.34404

**Published:** 2025-10-01

**Authors:** Roshanak Saghebi, Seyyedeh Zahra Adabinia, Morteza Mojahedi, Jamal Rezaei Orimi, Zahra Aghabeiglooei

**Affiliations:** ^1^Traditional Medicine and History of Medical Sciences Research Center, Health Research Institute, Babol University of Medical Sciences, Babol, Iran; ^2^Dr. Nourani Vesal Museum and Scientific and Cultural Documentation Center, Shiraz University of Medical Sciences, Shiraz, Iran; ^3^Department of History of Medical Sciences, School of Iranian Medicine, Babol University of Medical Sciences, Babol, Iran; ^4^Research Center for Traditional Medicine and History of Medical Sciences, Mazandaran University of Medical Sciences, Sari, Iran; ^5^Traditional Medicine Clinical Trial Research Center, Shahed University, Tehran, Iran; ^6^Department of Traditional Medicine, School of Persian Medicine, Shahed University, Tehran, Iran

**Keywords:** Eye disease, First Pahlavi era, Trachoma, Medical history, Ophthalmology

## Abstract

Trachoma was one of the most prevalent infectious illnesses in Iran during the first Pahlavi era, causing widespread suffering and blindness. This research aims to investigate the background of dealing with trachoma in Iran from 1925 to 1941. It seeks to answer questions about trachoma by using descriptive and analytical methods, drawing on documents and evaluating historical data. The results of the research show that during the first Pahlavi era, due to the serious health situation, there was a pressing need to address trachoma and take necessary steps. The government worked in a number of sectors to tackle the trachoma epidemic, including preventive measures, establishment of medical centers, budgetary approval and legislation approval, activities of ophthalmologist, and scientific research. Trachoma treatment involves general strengthening, local treatment (drugs, surgery, electrotherapy), and treatment of complications. The most important drug used to combat trachoma was *sulfate de cuivre* (copper sulfate). Lack of organized institutions, failure to correctly diagnose the disease, infrastructural issues, and centralist government policies, insufficient financial resources, and inadequate budget allocation were significant obstacles against eradicating and fighting the disease. From 1925 to 1941, the Iranian government made significant efforts to combat trachoma, but limited resources and infrastructure hindered long-term effectiveness. However, these early efforts laid the groundwork for future public health programs.

## Introduction

 Trachoma is one of the main causes of blindness in the world and the most dangerous infectious eye disease, caused by “*Chlamydia trachomatis*”.^1,2^ Its mention in ancient Egyptian medical texts and its surgical treatment in the law of Hammurabi in Mesopotamia show its ancient history in human life.^3^ Old documents refer to Chinese therapies (2600 B.C.) and Hippocratic Corpus, as well as the works of several physicians, including Celsus (1st century A.D.), Dioscorides (40-91 A.D.), and Galen (129-216 A.D.).^4^

 This disease was known among Iranian physicians in earlier times, and there had been signs of its description and treatment in ancient books.^5^ However, with the beginning of the Qajar era and the widespread outbreak of infectious diseases,^6^ the first organized efforts to modernize the medical system and combat diseases like trachoma began.^7^ The establishment of Dar al-Funun in 1851 was a turning point in this regard. With the arrival of European doctors and the teaching of modern medicine at this center, new diagnostic and treatment methods were introduced to the country.^8^ These doctors, upon observing the widespread prevalence of diseases such as trachoma, began efforts to educate and treat it.^9^ However, due to infrastructural limitations, lack of public awareness, and absence of a centralized public health system, these measures during the Qajar period were mostly individual and confined to urban centers.^10^

 During the first Pahlavi period (1925–1941), trachoma, as an infectious eye disease, was widespread in many regions of Iran, especially in rural and deprived areas.^11^ Due to lack of safe drinking water, inadequate sewage, and poor personal hygiene, this disease spread rapidly among people and, in severe cases, led to blindness. The spread of trachoma during this period was a major public health problem, and a large number of people, especially children, lost their sight. For this reason, the first Pahlavi government initiated measures to fight this disease, which included the establishment of medical centers, public health education, and drug distribution.^12^

 The failure to carry out comprehensive and independent research on trachoma during this period,^3,5,13,14^ and the importance of explaining Iran’s experience in eradicating and combating trachoma for the benefit of countries with a high prevalence of the disease, highlight the necessity of conducting this research. According to the nature of the topic, the research method is descriptive and retrospective historical research, which aims to answer the research questions about trachoma by using documentary and library sources.

 This research is an effort, based on the available evidence, to examine the prevalence rate, contexts, and causes of the spread of trachoma in Iran, the actions taken to this disease, such as how it was diagnosed and treated, how it was managed in schools, the implementation of urban reforms and improvements in environmental health, the conduct of scientific research, and finally, an explanation of the existing problems in the path toward eradicating and combating trachoma. The purpose of this research is to examine the history of efforts to combat trachoma in Iran from 1925 to 1941.

## Trachoma Prevalence Rate

**Figure 1:**
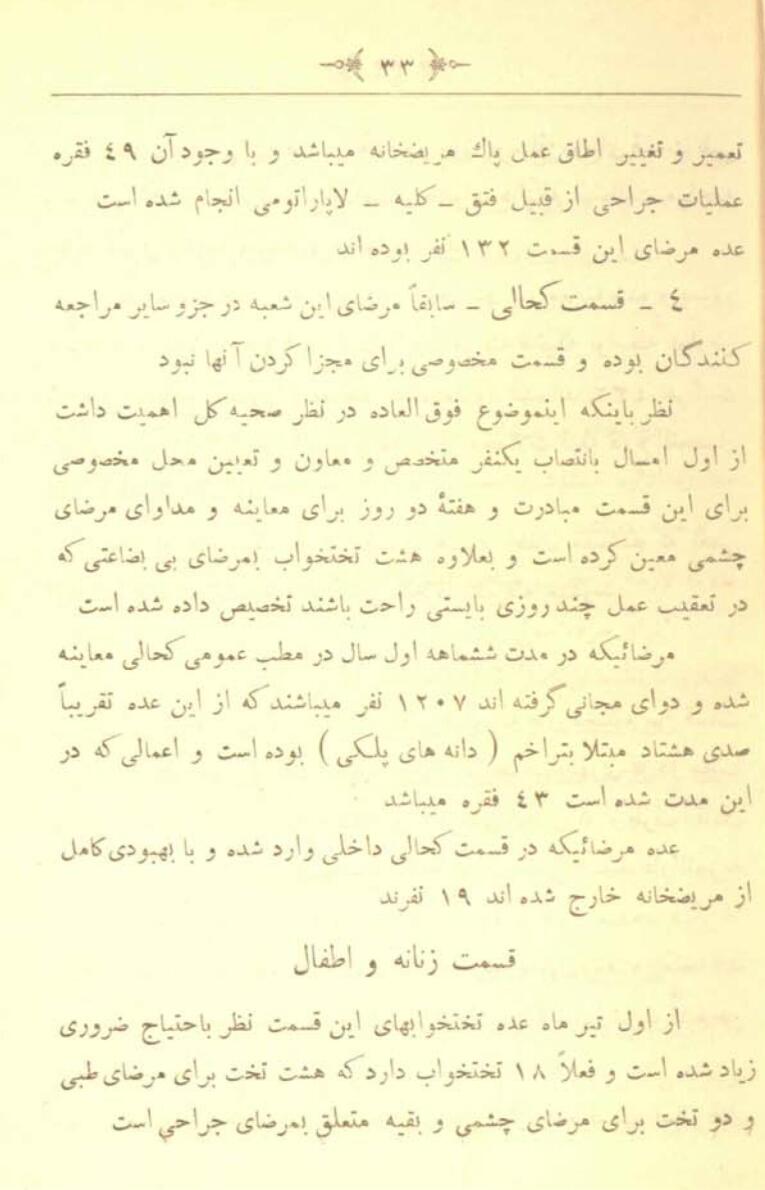
Report of the Health Department to the Government in 1926 about the Ophthalmology Department of the Waziri Hospital and the prevalence of trachoma.^17^

 Independent research and complete and accurate statistics about the spread of trachoma in all parts of Iran during the first Pahlavi period are not available. Trachoma was one of the most widespread infectious diseases and the most common cause of blindness during this time across Iran.^15^ At the beginning of the first Pahlavi period, about 33.2% of the Iranian population was suffering from trachoma, and it was the primary cause of blindness in 40% (12,450 people) of the blind population in Iran.^16^ In 1926, out of 1207 patients examined in a government hospital, about 80% were diagnosed with trachoma^17^ (Figure 1). Trachoma had spread in some southern cities, such as Dezful and Shushtar, due to the presence of cellars in houses, the absence of sewage wells, and poor sanitary conditions.^18^

 In 1934, Dr. General Colonie, head of the National Health Service, prepared a report on the status and spread of trachoma in Iran. The statistics revealed that trachoma was present in all regions of the country, with the highest prevalence in Khuzestan, Kerman, and the Persian Gulf. The infection rate was 68% in southwest Tehran, 61.9% in Jiroft, 47.29% in Bandar Lengeh, and 67.27% in Abadan.^19^ Dr. Fathullah Farahi reported that the prevalence of trachoma was 45% in Bushehr and 50% in Bandar Abbas (Table 1).^20^ By 1938, the majority of residents in Dezful were affected by trachoma and faced a severe shortage of ophthalmologists.^18^

**Table 1:** Trachoma Prevalence Statistics from Medical Centers in Selected Iranian Cities (1934–1936)^20,21^

**City**	**Reporting Location**	**Time Interval**	**Number of Trachoma Cases**
Boushehr	Eye Clinic	Dec 1934 - Jul 1935	1413 persons
Ahvaz	Eye Clinic, Municipal Office	Feb 1935 - May 1936	1683 persons
Shiraz	Eye Clinic, Municipal Office	Feb 1935 - Jul 1935	151 persons
Tehran	Rhazi Hospital	Jul 1934 - Dec 1934	5585 persons
Chaharmahal	Chaharmahal Hospital	Aug1934 - Sep 1934	334 persons

## Backgrounds and Causes of the Spread of Trachoma Disease

 One of the main causes of the spread of trachoma was the poor condition of the public health system at the beginning of the first Pahlavi period.^22^ Treatment methods were outdated, and modern medicine lacked the necessary infrastructure to prevent and treat diseases, especially infectious diseases. The inadequate health standards, particularly along roads, and the absence of medical facilities such as medical centers and trained Iranian doctors, can be considered as major contributors to the spread of this disease.^23^ Sewage and human waste often flowed through public passages, as documented in reports by the Khuzestan military sponsor,^24^ Akhgar magazine from Isfahan in 1928,^25^ and the 1934 report by Dr. Colonie, head of the National Health Department.^26^

 Lack of advancement in ophthalmology knowledge and the absence of specialized clinics and trained specialists were also key factors in the prevalence of trachoma during the early Pahlavi period. In Tehran, many patients with eye diseases such as trachoma were hospitalized in general wards due to the lack of dedicated ophthalmology units. The ophthalmology departments in government hospitals (including Vaziri and women’s hospitals) were largely inactive, with eye patients examined only two days a week, and hospitalized only if surgery was required.^11,17,27^ Other contributing factors to trachoma included weather conditions, altitude, geographical location, heat, dust, atmospheric irritations, flies, poverty, overcrowded living environment, scratches on the eyelid conjunctiva, syphilis, and tuberculosis. While climatic conditions did not directly cause trachoma, the hot and dry climate of southern Iran significantly influenced its spread.^28^ Ports of southern Iran and Khuzestan were particularly affected due to poor hygiene and environmental conditions.^29^ The disease was also less prevalent in high-altitude and colder regions compared to low-lying tropical areas.^27^

## How to Diagnose and Treat Trachoma

###  Diagnosis

 During the first Pahlavi period, general practitioners and ophthalmologists were responsible for diagnosing and treating trachoma. Ophthalmologists like Prof. Chams emphasized thorough patient examination for accurate diagnosis. The method involved turning the upper eyelid inside out so that its cartilage and conjunctiva could be examined, and the conjunctival gyrus fully exposed. Doctors also considered symptoms such as sabella (chronic vascular complication of Trachoma), related complications, absence of parotid gland swelling, presence of granular glands on the cartilage conjunctiva, healing signs, and the course of the disease. Complications of trachoma, including drooping eyelids, narrowing of the eyelids, inward turning of the eyelids, trichiasis, and ectropion played an important role in the diagnosis. Additionally, identifying complications of trachoma on the conjunctiva, such as symblepharon and xerosis, and on the cornea, such as pannus, corneal ulcers, and corneal staining, was decisive in determining the stage of the disease.^20,28^

###  Treatment

 Ophthalmologists like Professor Chams outlined three important principles in the treatment of trachoma: the first was general treatment, which aimed to strengthen the patient using general body tonics (both chemical and natural) such as arsenic, iron, cold water baths, seawater, mineral waters, and travel to summer resorts. Lymph node treatment was also included. The second is the local treatment of trachoma, which involved medication, surgery, and electrotherapy to promote recovery. The third is treatment of complications, addressing the aftereffects of the disease. This included injecting cacodylate disodium and glycerophosphate to improve the patient’s vitality.^20^ In the book Eyelid Boils, Dr. Colonie provided guidance for physicians on the simplified treatment of trachoma. He advised that doctors who were not ophthalmology specialists should prescribe easy-to-use but effective medications, especially in the early stages. Painful procedures such as shaving by cutting were discouraged, as they could repel patients and prevent follow-up visits. He also recommended avoiding the treatment of severe side effects, which should be left to specialists.^19^

###  Medication

**Figure 2:**
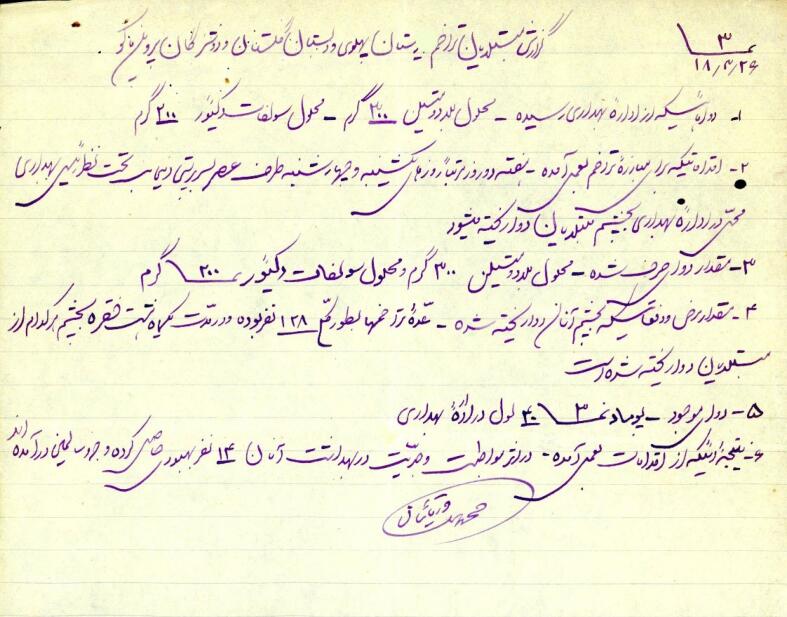
Report of Trachoma Sufferers in Mako Schools in 1939 and the Use of Methylene ‘Blue Solution” and Copper Sulfate in the Treatment of Students.^31^

 The treatment of trachoma involves the use of various drugs, including mercury cyanide (cyanide de mercure) solution, copper sulfate (sulfate de cuivre) drug, Argirel solution, methylene blue (bleu de methylene) ointment, and Iodoform ointment.^19,29^ Mercury cyanide solution was used in acute cases, while copper sulfate was applied after the acute phase. The most important medication was copper sulfate, also known as Kaat-e-kabood in Persian, which was prepared using a specific formula at the Institute of Eye, Ear, and Nose Diseases (Figure 2). Dr. Nosratollah Khan Bastan (1903–1986), developed a combination of copper sulfate and sublimate, which was used in municipal hospitals and school clinics. However, this method was not widely recommended due to its painful nature and unsatisfactory results. The process involved gently massaging the trachoma bumps to open them, and then using the medicated cotton to smooth the inside of the eyelid.^30^

**Figure 3:**
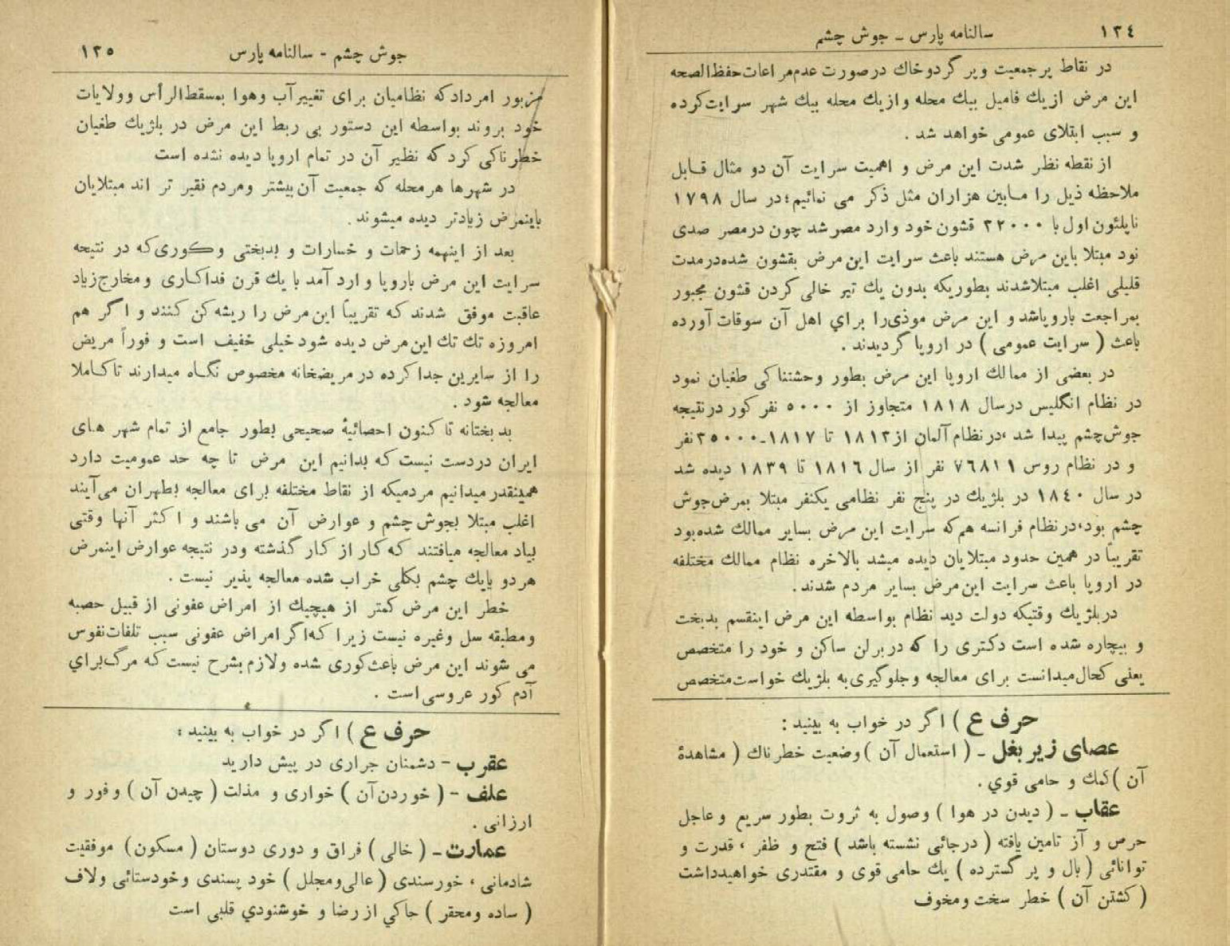
Note Published in the Pars Almanac in 1934 Regarding the Method of Treating Trachoma.^32^

 According to the note published in Pars Almanac in 1934 about trachoma, brief instructions were provided for its treatment. These included the use of sunglasses to reduce light sensitivity, warm water poultices for dropsy and severe pain, and eye washing with a 1% silver nitrate (nitrate d’argent) solution or potassium permanganate to treat adhesions. Additionally, for affected children without access to a doctor, it was recommended that they wash their eyes four times a day with a 2% sodium borate solution, and apply two drops of a 1% silver nitrate solution in the morning and evening^32^ (Figure 3).

###  Surgery

 Dr. Bastan believed that trachoma treatment was lengthy and difficult, requiring multiple incisions and grooves to penetrate the tissue. He considered manual and surgical procedures the most effective methods for treating trachoma.^30^ Professor Chams, however, did not support surgical treatment for trachoma pimples, as he believed surgery damaged the conjunctival and corneal cells, reducing their resistance. Instead, he treated trachoma pimples with copper sulfate solution and removed hard boils using electrocoagulation. Dr. Chams also opposed burning trachoma boils with a needle or using galvanopuncture.^20^ In 1936, patients suffering from corneal spots caused by trachoma complications were sent to Farabi Hospital, where a corneal transplants were performed by Professor Chams. Various trachoma complications, such as corneal ulcers, keratitis (Corneal inflammation) and trichiasis (Eyelashes growing towards the cornea) were treated in hospitals across different cities.^27,30^

###  Diathermocoagulation

 Another innovative method used to treat trachoma in the first Pahlavi period, following the establishment of Tehran University, was diathermy treatment, or electrical coagulation of trachoma boils, which differed from traditional burning techniques. In this method, only the needle was inserted into the boils, and the tissue was coagulated.^33^ Professor Chams, along with Professor “Jacques Relay”, conducted studies on trachoma and diathermy at the Ophthalmological Assembly of Lyon, France, and published their findings in a scientific journal in 1930.^20,34^ They stated that electrocoagulation was performed only on the bumps. After returning to Iran, they started the treatment of trachoma with their treatment method called diathermocoagulation and successfully treated a large number of trachoma patients by coagulating the entire eyelid without complications. In this procedure, unipolar diathermy was used for mild cases and bipolar diathermy for severe ones. The process began with injecting the nocaine solution into the eyelid cartilage and applying a drop of cocaine into the eye. Then, the operation was initiated using a needle. After adjusting the electric current intensity (200 to 300 milliamps), the needle was placed on the conjunctiva and trachoma seeds. After the procedure, argyrol was applied to the eye, and an antiseptic solution was used on the inner eyelid in the following days.^33^

## Measures Taken in the Fight Against Trachoma

###  Preventive Measures

 As part of the first Pahlavi government’s efforts to expand and improve the health system,^35,36^ several actions were taken to combat trachoma across multiple sectors (Table 2). In the prevention sector, the government promoted education and public awareness, and tried to prevent the spread of the disease by talking about it in the public arena and using cultural tools such as publishing books and newspapers, holding training classes, advertising, and films.^3,5^

**Table 2:** Review of Preventive Measures, Treatment Strategies, Scientific Activities, and Legislative Actions in Addressing Trachoma

**Preventive Measures**	**Treatment Measures **	**Scientific Activities**
•Individual, family, social, national, and international prevention •Educating personal and social hygiene •Emphasizing the use of soap and water •Preventing students from attending school^19^ •Organizing educational sessions on infectious diseases, including trachoma, by the Department of Education •Holding health conferences by the Ministry of Education and Endowments regarding trachoma in schools and involving teachers, administrators, students, and school staff along with their families •Allocating sections of newspapers to trachoma and preventive health measures (Figure 4) •Announcements and cinema films •Training school teachers •Examining students in schools and educational centers^30^	•Referring patients with trachoma to public or private general medical practices and eye specialists^29^ •Increasing ophthalmic services in hospitals and private centers by establishing ophthalmology departments in governmental, state, women's, military, and educational hospitals^27^ •Establishing the Faculty of Medicine at the University of Tehran •Establishing Farabi Hospital •Establishing the Institute of Eye, Ear, Nose, and Throat Diseases^26,40^ •Expanding trachoma treatment clinics in most cities •Establishing the Trachoma Combat Society •Compilation of the "Trachoma Prevention and Treatment Charter" by the National Health Department, 1933^29^ (Figure 4)	•Compilation of the thesis "Trachoma" by Dr. Fathollah Farahi, 1937^20^ •Compilation of the book about "Trachoma” by Dr. Nasrollah Bastan, 1936^37^ •Compilation of the book "Eyelid Acne or Trachoma" by the Health Department, 1934^19^ •Compilation of the thesis "Trachoma and its Treatment by Electrocoagulation" by Dr. Fathollah Farahi, 1934^20^ •Compilation of the thesis "Trachoma and its Treatment in Syphilis" by Dr. Mohammad Reza Maskoub, 1937^38^ •Assigning medical students' theses to trachoma topics^20,38^ •Participating in international scientific congresses •Performing corneal transplantation surgery •Using the modern method of diathermocoagulation in trachoma patients^39^

**Figure 4:**
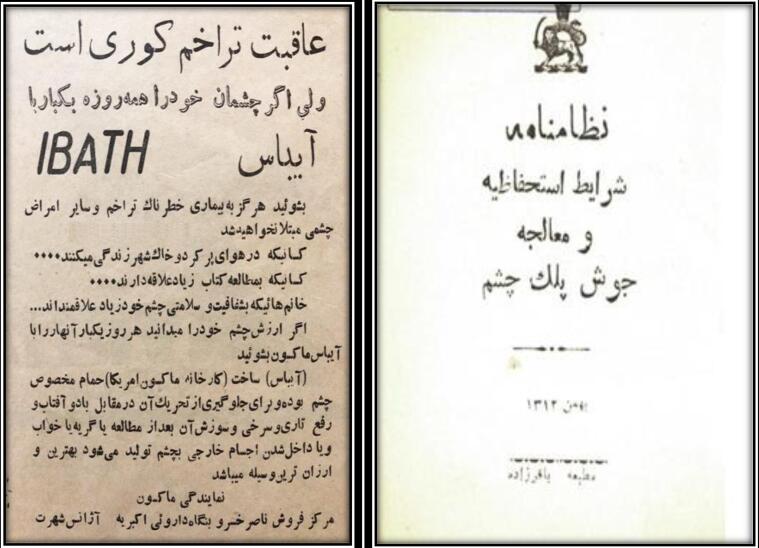
Left Side: Prevention of Trachoma Through the Use of Detergents and Hygiene Products in Newspapers. Right Side: Regulations for the Protection and Treatment of Eyelid Acne (Trachoma) in the Year 1933.^29^

## Establishment of Medical Centers

 In 1936, the Ministry of Health established trachoma clinics in most cities, while the Naft Company Hospital provided similar services in the southern regions of the country. In 1934, a dedicated ophthalmology hospital was created within Amin Hospital in Isfahan, under the leadership of Dr. Mirhsinkhan Miralaei. Additional governmental measures included:

- Treatment of patients by specialized physicians,
- Training of provincial health doctors,
- Distribution of anti-trachoma medications to health posts,
- Designation of Razi Hospital and Vaziri Hospital in Tehran as referral centers for trachoma patients.

These initiatives reflect the government’s commitment to expanding medical infrastructure and improving access to specialized care for trachoma patients.^11,34,41^

## Approval of Laws and Budget Allocation

 Due to the significant impact of trachoma on public health, the issue was elevated to the legislative level. Governmental actions included:

- Approval in 1928 to send a student to Europe for ophthalmology training,^42^
- Allocation of funds in 1933 for trachoma control and medication distribution,^19^
- Importation of specialized drugs for trachoma treatment in 1933,^32^
- Budget approval in 1934 for the establishment of a dedicated trachoma treatment program,
- Enactment of legislation targeting infectious diseases such as cholera, plague, yellow fever, and neonatal conjunctivitis (including trachoma),
- Mandated reporting of suspected cases by physicians, midwives, family heads, guesthouse owners, and clergy.^19,24^

These legislative and financial measures underscore the government’s strategic approach to combating trachoma through both medical and regulatory frameworks.

## Ophthalmologist Activity

 In the meantime, Iranian ophthalmologists such as Professor Chams (1904-1996) had a great impact in eradicating trachoma.^27^ The important actions of Professor Chams included establishing ophthalmology in schools, establishing Farabi Hospital, approving a six-month ophthalmology course for medical students, teaching ophthalmology, performing the first corneal transplantation in Iran, and treating via diathermocoagulation.^39^ Through his efforts, Farabi Ophthalmology Hospital flourished in 1940 and joined the Faculty of Medicine.^43^ In 1936, Professor Chams requested assistance from Max Frelin, head of the World Ophthalmology Association, to send foreign doctors to Iran to combat trachoma in Dezful. This request was approved, resulting in the arrival of three Austrian ophthalmologists. Several Iranian ophthalmologists also worked in southern Iran for several years.^27^ One of the notable initiatives of ophthalmologists was the establishment of the “Association for Combating Trachoma” by Dr. Bastan and colleagues in Tehran. Some members of this association treated patients free of charge in their office and displayed the association’s free clinic sign on their doors.^28^

## Scientific Research

 Alongside efforts to combat trachoma, scientific research in this field began to emerge. In 1934, Dr. Fathullah Farhi devoted his medical treatise to “trachoma and its treatment by electrocoagulation,” which was one of the first scientific studies on trachoma following the establishment of Iran’s medical school.^20^ In 1936, Dr. Mohammad Reza Maskoub authored the treatise “Trachoma and its Treatment in Syphilis Patients,” covering topics such as the histology of healthy conjunctiva and trachoma, classification, symptoms, complications, diagnosis, and treatment of trachoma in separate chapters.^38^ That same year, Dr. Nusratullah Bastan conducted research on conjunctival diseases, particularly trachoma. He classified trachoma into primary, general, and healing categories, based on foreign ophthalmologists like Mack Callan.^29^ Professor Chams conducted extensive research on trachoma in rabbits and dogs, revealing that these animals have high resistance to the disease. To reduce their resistance, they were deprived of food for twelve days before trachoma was inoculated into their conjunctiva.¹⁴ Reconstruction of the administrative-health structure of the country, efforts to control diseases, building hospitals, establishing clinics, free treatment for the poor, increasing the number of doctors and nurses, and announcing public health regulations^44,45^ were among the measures that indirectly played an important role in dealing with trachoma.

## Problems in the Way of Eradication and Fighting against Trachoma

 The eradication and fight against trachoma in Iran faced challenges due to the lack of regular organizations for epidemiology and etiology studies. General statistics of trachoma patients were limited and had no real value. The Ministry of Health and schools’ statistics were problematic due to the limited presence of ophthalmologists in large cities and the lack of eye examination equipment in other cities. These statistics were collected only from patients visiting ophthalmology or general clinics.^28^

 According to some ophthalmologists, beyond the aforementioned issues, public reluctance and avoidance of medical examinations were major obstacles to obtaining accurate statistics on the disease.^46^ Additionally, due to insufficient funding and personnel, final-year medical students were tasked with inspecting and examining schools. Their limited clinical experience sometimes led to diagnostic errors, including the treatment of healthy individuals instead of those with trachoma. Consequently, the resulting statistics were often distorted.^22^ Some of the challenges were rooted in the country’s infrastructural deficiencies, compounded by the centralist policies of the government, particularly in provincial areas.^47,48^ Factors such as geographic inaccessibility, lack of transportation infrastructure, shortage of physicians, medications, and clinics, low public awareness, and inadequate health literacy hindered the government’s efforts to combat the disease. The report on the prevalence of trachoma in southern cities such as Dezful and southeastern regions like Sistan highlight the severity of the situation, particularly regarding limited access to medication.^18,49,50^

 Another challenge was correctly diagnosing the disease. Since the microbe was not yet discovered and information was scarce, trachoma was given different names based on its appearance or its similar symptoms to other types of conjunctivitis. As a result, each variation was thought of as a separate disease. Trachoma could also be misdiagnosed as other bumps that appear on the inner eyelid, such as lymphoid follicles, or even confused with glandular, follicular, and seasonal conjunctivitis.^51,52^

## Conclusion

 The study’s findings suggest that actions needed to be taken to combat the illness and necessary steps were implemented to contain and eradicate trachoma due to the severe circumstances surrounding the epidemic in the late Qajar and early Pahlavi periods. Trachoma was one of the most frequent illnesses in Iran resulting from a number of factors, including the country’s poor health state, lack of developed medical and health knowledge, dearth of specialist ophthalmology clinics, and shortage of highly qualified ophthalmologists.

 Consequently, the implemented measures, such as the establishment of the Tehran University Medical School and Farabi Hospital, the expansion of ophthalmic services in both public and private hospitals, the opening of a trachoma clinic, the publication of regulations on the prevention and treatment of trachoma, the approval of a budget for trachoma control, the establishment of ophthalmology departments in military and educational health facilities, an increase in the number of graduates in modern ophthalmology, the fight against trachoma in schools, and scientific research activities, played a significant role in the battle against trachoma.

 In this regard, the government endeavored to prevent the spread of trachoma through cultural tools such as publishing books and newspapers, conducting educational classes, advertising, and utilizing films, as well as policies such as providing free healthcare services. Governmental efforts to combat trachoma yielded relatively temporary results; this was due to the government’s lack of necessary infrastructure, budget, and human resources in the health and medical sectors. Consequently, it was unable to conduct extensive and serious educational and cultural campaigns against the disease. However, these actions laid the groundwork for more serious efforts in subsequent years.
